# Referenzauswertungen für die Schätzung von Prävalenz, Inzidenz und Mortalität Public-Health-relevanter Erkrankungen auf Basis von Routinedaten

**DOI:** 10.1007/s00103-023-03821-1

**Published:** 2024-01-08

**Authors:** Laura Krause, Lukas Reitzle, Steffen Hess, Thomas Ziese, Davis Adewuyi

**Affiliations:** 1 Abteilung für Epidemiologie und Gesundheitsmonitoring, FG 24 Gesundheitsberichterstattung, Robert Koch-Institut, General-Pape-Str. 62–66, 12101 Berlin, Deutschland; 2 Bundesinstitut für Arzneimittel und Medizinprodukte, Forschungsdatenzentrum Gesundheit, Bonn, Deutschland

**Keywords:** Sekundärdaten, Gesetzliche Krankenversicherung, Nicht-übertragbare Erkrankungen, Surveillance, Public-Health-Forschung, Secondary data, Statutory health insurance, Non-communicable diseases, Surveillance, Public health research

## Abstract

Die Routinedaten aller gesetzlich Krankenversicherten nach Datentransparenzverordnung (DaTraV-Daten) stellen eine vielversprechende Datenquelle für die wiederkehrende und zeitnahe Surveillance nicht-übertragbarer Erkrankungen (NCD) in Deutschland dar. Dabei hat sich gezeigt, dass ein hoher Bedarf für Referenzauswertungen besteht, die schnelle und regelmäßig wiederholbare Analysen zu wichtigen NCD ermöglichen. Vor diesem Hintergrund wurde „ReFern-01“ initiiert, ein gemeinsames Projekt vom Robert Koch-Institut (RKI) und dem Bundesinstitut für Arzneimittel und Medizinprodukte (BfArM). In Zusammenarbeit mit Expert:innen aus dem Bereich der Sekundärdatenanalyse und Versorgungsforschung wurden Referenzauswertungen zur Schätzung von Prävalenz, Inzidenz und Mortalität für wichtige Public-Health-relevante Erkrankungen erarbeitet. Zunächst wurden mittels einer Onlinebefragung 11 zentrale NCD ausgewählt und in Zusammenschau mit einer Literaturrecherche initiale Falldefinitionen erstellt. Diese wurden anschließend in einem virtuellen Workshop diskutiert und konsentiert. Die erstellten Referenzauswertungen (Analyseskripte) ermöglichen eine standardisierte Schätzung der genannten epidemiologischen Kennzahlen, die über die Zeit und regional vergleichbar sind. Neben der Bereitstellung der Ergebnisse werden die Skripte am BfArM für zukünftige Datennutzende zur Verfügung stehen. Mit dem Fernzugang zur Analyse der DaTraV-Daten, der derzeit am Forschungsdatenzentrum Gesundheit (FDZ Gesundheit) aufgebaut wird, können die Ergebnisse des Projekts ReFern die Surveillance von NCD stärken und Public-Health-Akteur:innen beispielsweise bei der Planung und Umsetzung von Maßnahmen der Gesundheitsförderung und Prävention auf Ebene von Bund, Ländern, Kreisen und Kommunen unterstützen.

## Abstract (en)

The routine data of all statutorily insured persons according to the Data Transparency Regulation (DaTraV data) represent a promising data source for the recurrent and timely surveillance of non-communicable diseases (NCDs) in Germany. Thereby, it has become apparent that there is a high demand for reference evaluations that enable quick and regularly repeatable analyses on important NCDs. Against this background, ReFern-01 was initiated, a joint project of the Robert Koch Institute (RKI) and the Federal Institute for Drugs and Medical Devices (BfArM). In collaboration with experts from the field of secondary data analysis and healthcare research, reference evaluations for estimating prevalence, incidence, and mortality for important public health-relevant diseases were developed. First, 11 central NCDs were selected by means of an online survey, and initial case definitions were created in conjunction with a literature review. These were then discussed and agreed upon in a virtual workshop. The created reference evaluations (analysis scripts) allow a standardized estimation of the mentioned epidemiological figures, which are comparable over time and regionally. In addition to providing the results, the scripts will be available at the BfArM for further analysis. Provided that remote access to the analysis of the DaTraV data is available in the future, the results of the ReFern project can strengthen the surveillance of NCDs and support public health actors, for example, in the planning and implementation of health promotion and prevention measures at the federal, state, county, and local levels.

## Hintergrund

Nicht-übertragbare Erkrankungen („non-communicable diseases“, NCD) zählen in Deutschland, aber auch weltweit zu den häufigsten Todesursachen [1]. Zudem tragen sie erheblich zur Einschränkung der in Gesundheit verbrachten Lebenszeit bei [2, 3]. Dies gilt insbesondere für Herz-Kreislauf-Erkrankungen, Krebserkrankungen, chronische Atemwegserkrankungen, Diabetes mellitus, aber auch für psychische Erkrankungen. Aus Sicht von Public Health sind die Vorbeugung von Krankheiten, die Verlängerung des Lebens und die Förderung der Gesundheit zentrale Ziele [4]. Für die Beobachtung der Bevölkerungsgesundheit (Public-Health-Surveillance) bedarf es epidemiologischer Kennzahlen wie Prävalenz, Inzidenz und Mortalität [2]. Die Weltgesundheitsorganisation (WHO) versteht unter Public-Health-Surveillance die kontinuierliche und systematische Erhebung, Zusammenführung und Analyse von gesundheitsbezogenen Daten und die zeitnahe Bereitstellung von Informationen an Entscheidungsträger:innen als Grundlage für die Planung, Umsetzung und Evaluation von Public-Health-Maßnahmen [5]. Mit Blick auf NCD hat sich der Begriff „Surveillance“ allerdings erst vor gut 10 Jahren international etabliert [6–8].

**Figure 1:**
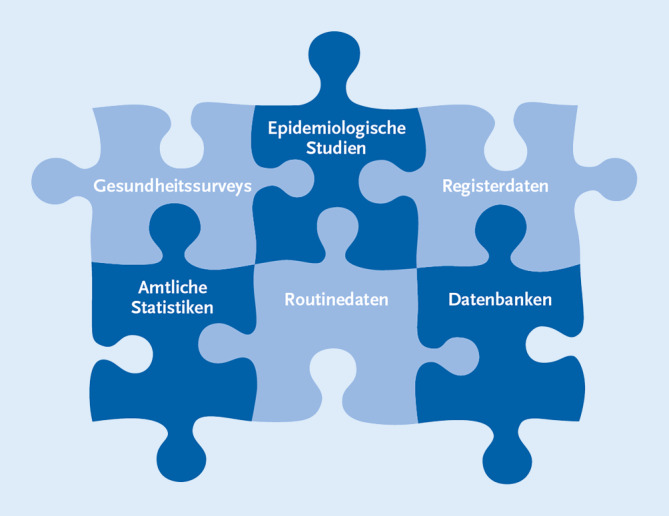

In Deutschland existiert bislang keine umfassende NCD-Surveillance und Informationen zur zeitlichen Entwicklung wichtiger NCD werden nicht systematisch erfasst [9], was die Entwicklung und Umsetzung von Präventionsmaßnahmen erschwert [10]. Vor einigen Jahren hat das Bundesministerium für Gesundheit die Abteilung 2 „Epidemiologie und Gesundheitsmonitoring“ des Robert Koch-Instituts (RKI) mit dem Aufbau einer Diabetes-Surveillance [11], einer Mental-Health-Surveillance [12, 13] sowie einer Surveillance umweltbasierter Einflussfaktoren auf die Entwicklung von Adipositas bei Kindern [14, 15] beauftragt. Darüber hinaus besteht ein registerbasiertes System zur epidemiologischen Surveillance des Krebsgeschehens [16]. Unter Einbezug dieser Surveillance-Systeme findet aktuell der Ausbau zu einer NCD-Surveillance statt [11]. Zukünftig wird auf Basis der ausgewählten NCD-Indikatoren die Gesundheitsberichterstattung (GBE) des Bundes über das Krankheitsgeschehen der Bevölkerung in Deutschland berichten [17]. Hierfür werden neben dem RKI-Gesundheitsmonitoring (Primärdaten) weitere Datenquellen benötigt (Abb. 1). Routinedaten (Sekundärdaten) aus dem Versorgungskontext gewinnen zunehmend an Bedeutung und ergänzen eine wiederkehrende und umfassende Beschreibung des Krankheitsgeschehens im Rahmen der geplanten NCD-Surveillance. Vorteile sind die routinemäßige Erfassung dieser Daten, der Ausschluss zentraler Fehlerquellen, wie zum Beispiel der Non-Response sowie der große Stichprobenumfang, der tief stratifizierte und regional kleinräumige Analysen in kurzen Zeitintervallen erlaubt [17].

**Figure 2:**
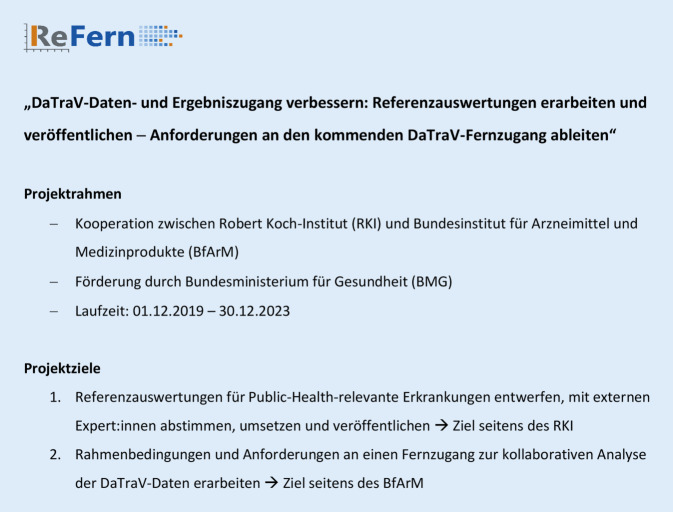

Bislang ist die Datenlandschaft aber stark fragmentiert und der Zugang für Forschende und Public-Health-Akteur:innen erschwert, wie das aktuelle Gutachten zur Resilienz im Gesundheitswesen des Sachverständigenrats Gesundheit (SVR) zeigt [10]. Die Novellierung der §§ 303 a–e des Fünften Buches Sozialgesetzbuch (SGB V) und der Datentransparenzverordnung (DaTraV) erweitern die Möglichkeiten der Nutzung und Nutzbarkeit von Routinedaten der Gesetzlichen Krankenversicherung (GKV), auch DaTraV-Daten genannt. Mit dem Aufbau eines Forschungsdatenzentrums Gesundheit (FDZ Gesundheit) am Bundesinstitut für Arzneimittel und Medizinprodukte (BfArM) wird eine Struktur geschaffen, die eine verbesserte und schnellere Nutzbarkeit der DaTraV-Daten für Nutzungsberechtigte und eine breite Dissemination der Ergebnisse in Zukunft ermöglicht. Rückmeldungen von Nutzungsberechtigten an das FDZ haben ergeben, dass ein hoher Bedarf an standardisierten Referenzauswertungen besteht, die schnelle und regelmäßig wiederholbare Analysen zu wichtigen Public-Health-Erkrankungen ermöglichen. Vor diesem Hintergrund wurde „ReFern-01“ initiiert, ein gemeinsames Projekt von RKI und BfArM (Abb. 2).

Der vorliegende Beitrag gibt einen Einblick in das Projekt ReFern-01 und berichtet über die Auswahl der Erkrankungen, die Festlegung der Falldefinitionen zur Berechnung von Prävalenz, Inzidenz und Mortalität sowie über die Erstellung der Referenzauswertungen. Aufgrund des derzeit noch fehlenden Fernzugangs zur Analyse der DaTraV-Daten steht eine Umsetzung allerdings noch aus.

## Krankheiten im Fokus

**Figure 3:**
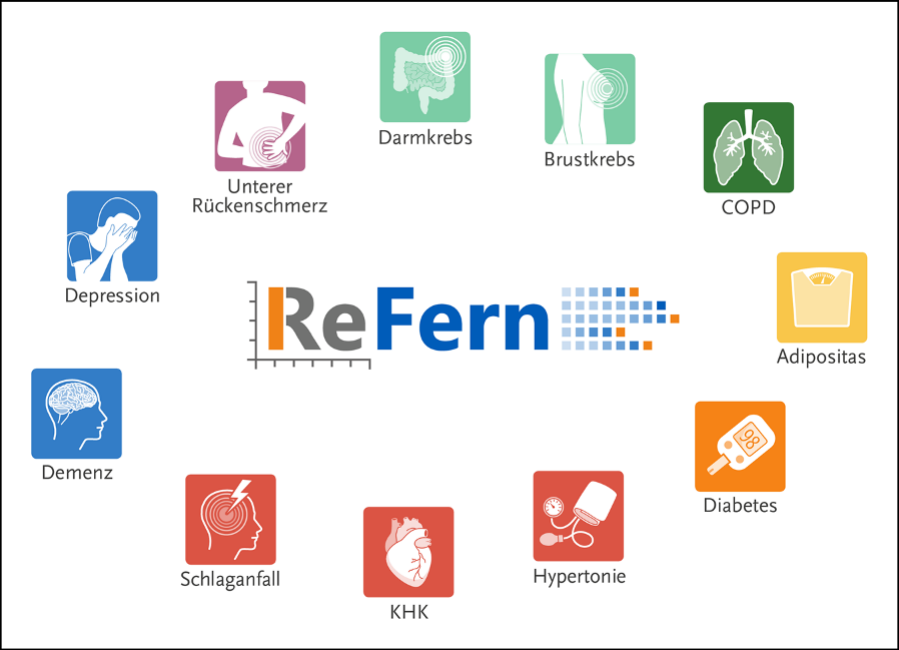

In ReFern stehen 11 Krankheiten mit hoher Public-Health-Relevanz im Fokus (Abb. 3). Die Auswahl der Erkrankungen erfolgte auf Basis der Krankheitslast gemäß der Global-Burden-of-Disease-Studie für Deutschland [18] und in Abstimmung mit laufenden Projekten am RKI, wie der Diabetes-Surveillance [11], der Mental-Health-Surveillance [12], der nationalen Krankheitslaststudie „BURDEN 2020 – Die Krankheitslast in Deutschland und seinen Regionen“ [19] sowie in Abstimmung mit dem Zentrum für Krebsregisterdaten (ZfKD; [20]). Darüber hinaus wurde bei der Auswahl der Erkrankungen auch auf die Umsetzbarkeit der Auswertung in den DaTraV-Daten geachtet und es wurden bereits existierende Publikationen zu Auswertungen in Routinedaten berücksichtigt. Die Krankheitsauswahl und deren Operationalisierung in Routinedaten wurde anschließend mithilfe einer Onlinebefragung und eines virtuellen Workshops überprüft und konsentiert [21].

## Entwicklung der Falldefinitionen

Für die Auswertung von Prävalenz und Inzidenz sowie bei manchen Erkrankungen auch Mortalität wurden auf Basis der DaTraV-Daten Falldefinitionen entwickelt, welche die eingeschlossenen ICD (International Statistical Classification of Diseases and Related Health Problems)-Codes sowie deren Dokumentation im ambulanten und stationären Sektor beschrieben. Für die Falldefinitionen von Diabetes mellitus und Depression wurde auf die bereits konsentierten Definitionen zurückgegriffen, welche im Rahmen der Diabetes- bzw. Mental-Health-Surveillance am RKI im Austausch mit dem jeweiligen wissenschaftlichen Beirat entwickelt wurden [22]. Die Falldefinitionen zu den anderen Erkrankungen wurden in 3 Schritten entwickelt. Zunächst wurde eine Onlinebefragung mit nationalen Expert:innen durchgeführt. Hierbei wurden die Teilnehmenden auch gefragt, ob Interesse besteht, die Ergebnisse in einem Workshop anschließend zu diskutieren. Zeitgleich mit der Onlinebefragung erfolgte eine Literaturrecherche, wobei sowohl *PubMed* als auch graue Literatur nach Publikationen zu den ausgewählten Erkrankungen durchsucht wurden. Zuletzt wurden die Ergebnisse der Onlinebefragung in Zusammenschau mit den Ergebnissen der Literaturrecherche in einem virtuellen Workshop mit den Expert:innen, welche sich bereit erklärt hatten teilzunehmen, diskutiert und Falldefinitionen für die Auswertungen konsentiert. Im Folgenden werden die Onlinebefragung, die Literaturrecherche und der virtuelle Workshop näher erläutert.

## Onlinebefragung

Die Onlinebefragung zur Überprüfung der ausgewählten Erkrankungen war zweistufig aufgebaut: Zunächst gab es einen allgemeinen Teil zu Daten, Nutzung und Expertise der Expert:innen, anschließend folgte ein spezifischer Teil zu den Krankheitsdefinitionen. Die Einladung zur Onlinebefragung wurde an den Verteiler der „Arbeitsgemeinschaft Erhebung und Nutzung von Sekundärdaten“ (AGENS), den Verteiler des „Deutschen Netzwerks für Versorgungsforschung“, an einen Verteiler mit Expert:innen aus den Fachbeiräten des RKI sowie an Mitglieder der Arbeitsgemeinschaft Sekundärdaten am RKI versandt. Zudem wurde der Befragungslink auf der Projektseite im Internet platziert (www.rki.de/refern). Durchgeführt wurde die Onlinebefragung von Mitte April bis Ende Mai 2021. Insgesamt gab es 414 Link-Aufrufe und 116 Teilnahmen („Teilnahme“ entspricht mindestens einer Antwort).

**Figure 4:**
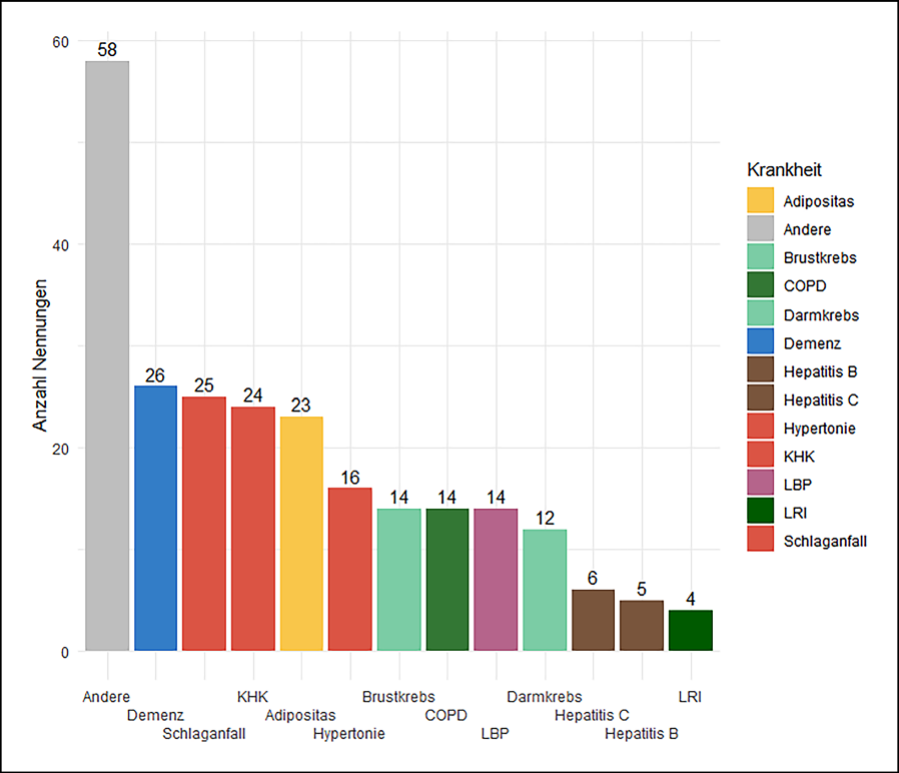

Der erste Teil der Befragung ergab, dass die Mehrheit der Teilnehmenden bereits Erfahrung mit der Auswertung von Daten der GKV (87 %) oder ambulanten Abrechnungsdaten (35 %) hatte. Die DaTraV-Daten wurden von 13 % der Teilnehmenden genutzt. Abb. 4 ist zu entnehmen, zu welchen Erkrankungen die Expert:innen über spezifische Expertise verfügten. Jeweils etwa ein Viertel der Teilnehmenden gab an, spezifische Expertise zu Demenz, Schlaganfall, koronarer Herzerkrankung (KHK) oder Adipositas zu besitzen. Weiterhin wurden häufig „andere Erkrankungen“ genannt (*n* = 58). Bei der Freitextangabe zu „andere Erkrankungen“ wurden Diabetes mellitus (26 Teilnehmende) und psychische Erkrankungen (10 Teilnehmende) am häufigsten angegeben.

Sofern eine spezifische Expertise für eine der zur Auswahl stehenden Erkrankungen vorlag, wurde die teilnehmende Person im zweiten Teil der Befragung zur Falldefinition der jeweiligen Erkrankung in Routinedaten befragt. Zunächst wurde erhoben, welche ICD-Codes für die Definition verwendet werden sollen und wie diese in den Daten dokumentiert sein müssen. Hierbei wurde nach ambulantem und stationärem Sektor unterschieden. So konnte im stationären Sektor angegeben werden, ob nur Hauptdiagnosen oder Haupt- und Nebendiagnosen verwendet werden sollen. Im ambulanten Sektor wurde erfragt, ob eine Dokumentation in einem Quartal (m1Q) oder in mindestens 2 Quartalen (m2Q) vorliegen soll und ob zusätzlich eine Arzneimittelverordnung oder andere Kriterien berücksichtigt werden sollen. Zusätzlich wurde erhoben, ob neben gesicherten Diagnosen auch weitere Qualifizierungen eingeschlossen werden sollen und ob nur spezifische ärztliche Fachgruppen als sinnvoll erachtet werden. Zuletzt wurde die Einschätzung zur Zeitspanne der diagnosefreien Vorlaufzeit zur Berechnung der Inzidenz erfragt.

## Virtueller Workshop zur Entwicklung von Referenzauswertungen

Im virtuellen ReFern-Workshop, der im Oktober 2021 stattgefunden hat, standen die Ergebnisse aus der Onlinebefragung und die zu entwickelnden Definitionen zu den ausgewählten wichtigen Public-Health-Erkrankungen im Fokus. Im Zuge der Vorbereitung des Workshops wurden *PubMed* und graue Literatur selektiv nach Publikationen zu Auswertungen von Prävalenz und Inzidenz in Routinedaten zu den ausgewählten Erkrankungen durchsucht. Die gefundenen Falldefinitionen wurden extrahiert und abgeglichen. Zunächst wurden die Ergebnisse zum allgemeinen Teil der Onlinebefragung vorgestellt. Anschließend fand die Diskussion der Falldefinitionen zu den spezifischen Erkrankungen in Teilgruppen statt:

1. Teilgruppe: KHK, Hypertonie und Schlaganfall,
2. Teilgruppe: Adipositas, Demenz und akuter unterer Rückenschmerz,
3. Teilgruppe: chronisch obstruktive Lungenerkrankung (COPD), Brust- und Darmkrebs.

Insgesamt haben rund 40 Expert:innen mit breiter Expertise im Bereich der Sekundärdatenanalysen und Versorgungsforschung an dem Workshop teilgenommen. Bei der anschließenden Erstellung der Krankheitsdefinitionen wurden die Anmerkungen von den Expert:innen aus dem Workshop berücksichtigt. Hierzu wurde eine initiale Krankheitsdefinition basierend auf der Onlinebefragung und der Literaturrecherche erstellt. Zumeist gab es hinsichtlich der ICD-Codes für alle Erkrankungen große Übereinstimmungen. Die Art der Dokumentation der ICD-Codes variierte jedoch teilweise in Abhängigkeit von der Erkrankung und der verwendeten Datenquelle. Diese wurde dann eingehend mit den Expert:innen diskutiert, mit besonderem Augenmerk auf Abweichungen zwischen den Definitionen. Darüber hinaus fand, wie von einigen Workshop-Teilnehmenden gewünscht, ein Abgleich mit den Krankheitsdefinitionen aus BURDEN 2020 [19], dem morbiditätsorientierten Risikostrukturausgleich (Morbi-RSA; [23]) und dem ZfKD [20] statt. Tab. 1 zeigt die konsentierten Krankheitsdefinitionen zu Schlaganfall, KHK, Hypertonie, Diabetes, Adipositas, Depression, Demenz, akutem unteren Rückenschmerz, COPD, Brust- und Darmkrebs. Zusätzlich wurden Sensitivitätsanalysen für die ausgewählten Erkrankungen durchgeführt, um die Bedeutung einzelner Kriterien der Falldefinition auf die Schätzung von Prävalenz oder Inzidenz zu überprüfen.

**Table 1:** 

***Schlaganfall***	
ICD-Codes	I60 Subarachnoidalblutung I61 Intrazerebrale Blutung I62 Sonstige nichttraumatische intrakranielle Blutung I63 Hirninfarkt I64 Schlaganfall, nicht als Blutung oder Infarkt bezeichnet
Periodenprävalenz	1‑Jahres- und 10-Jahres-Prävalenz
Dokumentationsvariante	m1Q HD
Medikation	–
Weitere Qualifizierung	–
Diagnosefreie Vorlaufzeit (Inzidenz)	1 Jahr
Sensitivitätsanalyse	1. Vorlaufzeit: 1 Jahr vs. 6 Monate vs. keine Vorlaufzeit 2. Differenzierung ischämischer Schlaganfall (I63, I64) vs. hämorrhagischer Schlaganfall (I60–I62) 3. Bedeutung von I64.– 4. Vergleich mit Burden-Definition [35]
***Koronare Herzerkrankung (KHK)***	
ICD-Codes	I20 Angina pectoris I21 Akuter Myokardinfarkt I22 Rezidivierender Myokardinfarkt I23 Bestimmte akute Komplikationen nach akutem Myokardinfarkt I24 Sonstige akute ischämische Herzkrankheit I25 Chronische ischämische Herzkrankheit
Periodenprävalenz	1‑Jahres-Prävalenz
Dokumentationsvariante	m2Q amb. (G, Z) oder m1Q HD
Medikation	–
Weitere Qualifizierung	Gesichert und Zustand nach (amb.)
Diagnosefreie Vorlaufzeit (Inzidenz)	1 Jahr
Sensitivitätsanalyse	1. Berücksichtigung Anteil „Zustand-nach“-Diagnosen: „Zustand nach“ vs. ohne „Zustand nach“ 2. Akute (I20–I24) vs. chronische Krankheitsverläufe (I25) 3. Bedeutung von m1Q ND stat. 4. Vorlaufzeit: 1 Jahr vs. 2 Jahre 5. Vergleich mit Burden-Definition [35]
***Hypertonie***	
ICD-Codes	I10 Essentielle (primäre) Hypertonie I11 Hypertensive Herzkrankheit I12 Hypertensive Nierenkrankheit I13 Hypertensive Herz- und Nierenkrankheit I15 Sekundäre Hypertonie
Periodenprävalenz	1‑Jahres-Prävalenz
Dokumentationsvariante	m2Q amb. (G) oder m1Q HD
Medikation	–
Weitere Qualifizierung	–
Diagnosefreie Vorlaufzeit (Inzidenz)	1 Jahr
Sensitivitätsanalyse	1. Bestimmung Anteil sekundäre Hypertonie: I15 vs. I10–I15 2. m2Q amb. vs. m2Q amb. + m1Q HD vs. m2Q amb. + m1Q HD + m1Q ND 3. Vorlaufzeit: 1 Jahr vs. 2 Jahre
***Diabetes mellitus***	
ICD-Codes	E10 Primär insulinabhängiger Diabetes mellitus (Typ-1-Diabetes) E11 Nicht primär insulinabhängiger Diabetes mellitus (Typ-2-Diabetes) E12 Diabetes mellitus in Verbindung mit Fehl- oder Mangelernährung (Malnutrition) E13 Sonstiger näher bezeichneter Diabetes mellitus E14 Nicht näher bezeichneter Diabetes mellitus
Periodenprävalenz	1‑Jahres-Prävalenz
Dokumentationsvariante	m2Q amb. (G) oder m1Q HD/ND stat.
Medikation	–
Weitere Qualifizierung	–
Diagnosefreie Vorlaufzeit (Inzidenz)	1 Jahr
Sensitivitätsanalyse	1. Typ-1-Diabetes (E10) vs. Typ-2-Diabetes (E11); Zusatzanalyse zur Typendifferenzierung unter Verwendung von ICD-Codes, Alter und Medikation 2. Vorlaufzeit: 2 Jahre vs. 1 Jahr
***Adipositas***	
ICD-Codes	E66 Adipositas
Periodenprävalenz	1‑Jahres-Prävalenz
Dokumentationsvariante	m2Q amb. (G)
Medikation	–
Weitere Qualifizierung	–
Diagnosefreie Vorlaufzeit (Inzidenz)	2 Jahre
Sensitivitätsanalyse	1. E66 vs. E65–E68 2. m1Q stat. HD/ND für E66, E65–E68 3. Vorlaufzeit: 2 Jahre vs. 1 Jahr 4. m2Q amb. vs. m2Q amb. + m1Q HD vs. m2Q amb. + m1Q HD + m1Q ND
***Depression***	
ICD-Codes	F32 Depressive Episode F33 Rezidivierende depressive Störung (ohne 33.4) F34.1 Dysthymia
Periodenprävalenz	1‑Jahres-Prävalenz
Dokumentationsvariante	m1Q amb. (G) oder m1Q HD/ND
Medikation	–
Weitere Qualifizierung	–
Diagnosefreie Vorlaufzeit (Inzidenz)	–
Sensitivitätsanalyse	1. m2Q amb. (G) vs. m1Q amb. (G) 2. nur ambulant vs. stationär UND ambulant 3. Bedeutung Einbezug Dysthymia (F34.1) 4. Unterscheidung Schweregrad der Depression 5. Vergleich mit Burden-Definition [35]
***Demenz***	
ICD-Codes	F00 Demenz bei Alzheimer-Krankheit F01 Vaskuläre Demenz F02 Demenz bei anderenorts klassifizierten Krankheiten F03 Nicht näher bezeichnete Demenz F05.1 Delir bei Demenz G23.1 Progressive supranukleäre Ophthalmoplegie (Steele-Richardson-Olszewski-Syndrom) G30 Alzheimer-Krankheit G31.0 Umschriebene Hirnatrophie G31.82 Lewy-Körper-Krankheit
Periodenprävalenz	1‑Jahres-Prävalenz
Dokumentationsvariante	m2Q amb. (G) oder m1Q stat. HD/ND
Medikation	–
Weitere Qualifizierung	–
Diagnosefreie Vorlaufzeit (Inzidenz)	2 Jahre
Sensitivitätsanalyse	1. Vorlaufzeit: 2 Jahre vs. 1 Jahr vs. mehr als 2 Jahre 2. Ohne F05.1 Delir bei Demenz 3. Ohne m1Q ND 4. Vergleich mit Burden-Definition [35]
***Akuter unterer Rückenschmerz***	
ICD-Codes	M54.3 Ischialgie M54.4 Lumboischialgie M54.5 Kreuzschmerz
Periodenprävalenz	1‑Jahres-Prävalenz
Dokumentationsvariante	m1Q amb. (G) oder m1Q HD/ND stat.
Medikation	–
Weitere Qualifizierung	–
Diagnosefreie Vorlaufzeit (Inzidenz)	1 Jahr
Sensitivitätsanalyse	1. m2Q amb. (G) 2. M54.3, M54.4, M54.5 ohne M40–M54 Rest 3. Vorlaufzeit: 2 Jahre vs. 1 Jahr
***Chronisch obstruktive Lungenerkrankung (COPD)***	
ICD-Codes	J41 Einfache und schleimig-eitrige chronische Bronchitis J42 Nicht näher bezeichnete chronische Bronchitis J43 Emphysem J44 Sonstige chronische obstruktive Lungenkrankheit
Periodenprävalenz	1‑Jahres-Prävalenz
Dokumentationsvariante	m2Q amb. (G) oder m1Q HD/ND stat.
Medikation	–
Weitere Qualifizierung	–
Diagnosefreie Vorlaufzeit (Inzidenz)	2 Jahre
Sensitivitätsanalyse	1. J41–J44 (ohne J43) 2. Ohne Medikation vs. mit Medikation (H02AB, R03AC, R03AK, R03AL, R03BA, R03BB, R03CC, R03DA, R03DX) 3. Vergleich mit Burden-Definition [35]
***Brustkrebs***	
ICD-Codes	C50 Bösartige Neubildung der Brustdrüse (Mamma) D05.1 Carcinoma in situ der Milchgänge
Periodenprävalenz	10-Jahres-Prävalenz
Dokumentationsvariante	m2Q amb. (G) oder m1Q HD/ND stat.
Medikation	–
Weitere Qualifizierung	*Perspektivisch*: OPS und Medikation (Aromatasehemmer, Tamoxifen)
Diagnosefreie Vorlaufzeit (Inzidenz)	Mehr als 2 Jahre
Sensitivitätsanalyse	1. Berücksichtigung Anteil „Zustand-nach“-Diagnosen: Zustand nach vs. ohne Zustand nach (Prävalenz) 2. 10-Jahres-Prävalenz vs. 5‑Jahres-Prävalenz 3. Vorlaufzeit: 1 Jahr, 2 Jahre, 5 Jahre 4. Vergleich mit Burden-Definition [35]
***Darmkrebs***	
ICD-Codes	C18 Bösartige Neubildung des Kolons C19 Bösartige Neubildung am Rektosigmoid, Übergang C20 Bösartige Neubildung des Rektums
Periodenprävalenz	10-Jahres-Prävalenz
Dokumentationsvariante	m2Q amb. (G) oder m1Q HD/ND stat.
Medikation	–
Weitere Qualifizierung	–
Diagnosefreie Vorlaufzeit (Inzidenz)	Mehr als 2 Jahre
Sensitivitätsanalyse	1. 10-Jahres-Prävalenz vs. 5‑Jahres-Prävalenz 2. Vorlaufzeit: 1 Jahr, 2 Jahre, 5 Jahre 3. Vergleich mit Burden-Definition [35] 4. *Perspektivisch*: Diagnose m2Q amb. (G) im weiteren Verlauf mit/ohne OPS

*ICD* International Statistical Classification of Diseases and Related Health Problems, *m1Q* mindestens 1 Quartal,* m2Q* mindestens 2 Quartale,* HD* Hauptdiagnose, *ND* Nebendiagnose,* Z* Zustand nach der betreffenden Diagnose, *G* gesicherte Diagnose,* amb.* ambulant,* stat.* stationär, *OPS* Operationen- und Prozedurenschlüssel (amtliche Klassifikation zum Verschlüsseln von Operationen, Prozeduren und allgemein medizinischen Maßnahmen)

## Erstellung von Referenzauswertungen

Die Entwicklung und Veröffentlichung von Referenzauswertungen für die im Projekt betrachteten wichtigen NCD ist zentrales Ziel aufseiten des RKI (Abb. 2). Zur Ermittlung von Ergebnismengen, die sich aus den oben erwähnten Falldefinitionen zur jeweiligen Referenzauswertung ergeben, wurden innerhalb des Projekts Skripte erstellt, anhand derer SQL-Abfragen in der Datenbank des FDZ Gesundheit möglich sind. Die Programmierung der Skripte teilte sich dabei in mehrere Schritte und baute auf den vorangegangenen Skripten auf. So verwendeten alle Skripte die gleichen Ein- und Ausschlusskriterien für die Studienpopulation und erstreckten sich über den Zeitraum von 2009 bis 2018.

Im ersten Schritt wurde für jede Erkrankung – mit Ausnahme der Krebserkrankungen – die 1‑Jahres-Prävalenz für jedes Beobachtungsjahr definiert (Periodenprävalenz; Tab. 1). Aufbauend darauf konnten Inzidenz und Mortalität definiert werden. Da für die Krebserkrankungen eine 10-Jahres-Prävalenz (Periodenprävalenz) definiert ist, wurde für diese eine Prävalenz über den gesamten Beobachtungszeitraum geschätzt. Zur Schätzung der Inzidenz wurde zunächst eine Population unter Risiko definiert. Hierzu wurden Personen mit Dokumentation einer entsprechenden ICD-Diagnose der Falldefinition in einem definierten Zeitraum (diagnosefreie Vorlaufzeit) vor dem analysierten Berichtsjahr ausgeschlossen. Die diagnosefreie Vorlaufzeit variierte zwischen den verschiedenen Erkrankungen. Darüber hinaus wurden für die jeweiligen Krankheiten spezifische Sensitivitätsanalysen definiert, welche abschließend in das SQL-Skript integriert wurden.

Basierend auf den Falldefinitionen, die mit den externen Expert:innen erarbeitet wurden, wurden die Skripte zur Berechnung von Prävalenz und Inzidenz für alle hier betrachteten NCD sowie für Mortalität bei KHK, Diabetes, COPD, Brust- und Darmkrebs erstellt. Die Ergebnisse für alle Analysen werden aggregiert stratifiziert nach Alter und Geschlecht vom FDZ bereitgestellt. Für die Hauptanalysen werden die Ergebnisse zusätzlich nach Bundesland und regionaler sozioökonomischer Deprivation unter Verwendung des German Index of Social Deprivation (GISD; [24]) stratifiziert.

## Dissemination der Ergebnisse

Zukünftig sollen die Projektergebnisse auf verschiedensten Wegen verbreitet werden. Zum einen ist neben dem vorliegenden methodischen Beitrag eine Publikation im *Journal of Health Monitoring*, dem Publikationsorgan der GBE des Bundes, geplant (www.rki.de/johm). Darin soll eine Auswahl an Ergebnissen präsentiert werden. Zum anderen wird ein Dashboard erstellt, um die Projektergebnisse zu visualisieren und der Öffentlichkeit verfügbar zu machen. Das Dashboard stellt die im Projekt betrachteten 11 wichtigen NCD vor und visualisiert sie nach Geschlecht, Alter und Bundesland. Zusätzlich wird die zeitliche Entwicklung der Erkrankungen von 2009 bis 2018 aufgezeigt. Darüber hinaus haben die Projektergebnisse einen konkreten praktischen Nutzen auf nationaler Ebene: Die entwickelten SQL-Skripte zur Berechnung von Prävalenz, Inzidenz und Mortalität der im Projekt betrachteten 11 wichtigen NCD werden der allgemeinen (Fach‑)Öffentlichkeit zugänglich gemacht und können zur eigenen Nutzung herangezogen werden.

## Diskussion

Die Daten aller gesetzlich Krankenversicherten in Deutschland stellen eine Datenquelle mit großem Potenzial für die geplante NCD-Surveillance am RKI dar und ermöglichen die zeitnahe und wiederkehrende Schätzung von Prävalenz und Inzidenz wichtiger Public-Health-Erkrankungen. Im Austausch mit relevanten Expert:innen im Bereich der Sekundärdatenanalyse und Versorgungsforschung wurden Falldefinitionen für 11 Erkrankungen erarbeitet und die SQL-Skripte für Referenzauswertungen entwickelt. Diese bieten die Möglichkeit, über die Zeit und regional vergleichbare Schätzungen zur Prävalenz und Inzidenz bereitzustellen. Auf diese Weise werden gesundheitspolitische Akteur:innen in Deutschland regelmäßig über das Krankheitsgeschehen informiert.

In der Public-Health-Forschung werden Routinedaten von GKV-Versicherten schon länger für die Schätzung von Prävalenz und Inzidenz zentraler NCD, wie beispielsweise Diabetes [25, 26], KHK [27, 28] oder Demenz [29, 30], genutzt. Häufig kommen hierbei Daten einzelner Krankenversicherungen oder die bundesweiten ambulanten Abrechnungsdaten des „Zentralinstituts für die kassenärztliche Versorgung“ (Zi) zum Einsatz, da diese aktuell für statistische Analysen zugänglich sind. Mit den DaTraV-Daten steht zukünftig ein Datensatz bereit, der bundesweit alle GKV-Versicherten umfasst und die Daten des ambulanten und stationären Sektors vereint. Somit unterliegen die Daten einem geringeren „Kassenbias“ und ermöglichen die Durchführung longitudinaler und sektorübergreifender Analysen.

Die Falldefinitionen, die in der Befragung und Diskussion mit den Expert:innen für die in ReFern betrachteten Erkrankungen erstellt wurden, stimmten größtenteils mit den im Rahmen der Literaturrecherche gefundenen Falldefinitionen überein. Insbesondere hinsichtlich der einzuschließenden ICD-Codes gab es kaum Abweichungen. Im Detail traten dann aber doch Unterschiede auf: So wurden beispielsweise in manchen Analysen zusätzlich erbrachte Leistungen, dokumentiert als OPS-Code (Operationen- und Prozedurenschlüssel [OPS]) oder EBM-Ziffer (einheitliche Bewertungsmaßstab [EBM]), in der Falldefinition eingeschlossen [28]. Im Kontext unterschiedlicher Datenquellen führt dies zu Schätzungen, die je nach Falldefinition und Datenquelle variieren und nur begrenzt regional und über die Zeit vergleichbar sind. Referenzauswertungen können hier eine Lücke schließen, indem sie standardisierte Analysen, die wiederkehrend epidemiologische Kennziffern wie Prävalenz und Inzidenz sowie bei bestimmten Erkrankungen auch Mortalität bereitstellen. Das methodische Vorgehen wird klar und transparent beschrieben und die Analyseskripte werden am BfArM für weitere Datennutzende zur Verfügung gestellt. Gleichzeitig können diese Auswertungen für eine beschleunigte Antragsbearbeitung von allen Nutzenden der DaTraV-Daten verwendet werden, indem die entwickelten Auswertungsroutinen in Anträge mit weitergehenden Forschungsfragen eingebunden werden. Die Referenzauswertungen werden den Nutzwert der DaTraV-Daten deutlich erhöhen, da diese für weitere Akteur:innen aus dem Public-Health-Bereich zugänglich sind.

Mit Blick auf die DaTraV-Daten müssen einige *Limitationen* berücksichtigt werden. So sind GKV-Versichertenpopulationen trotz einer Vollerhebung nicht repräsentativ für die in Deutschland lebende Gesamtbevölkerung, da die Versicherten der privaten Krankenversicherung (PKV) nicht enthalten sind und sich beide Gruppen in ihrem Gesundheitszustand zum Teil voneinander unterscheiden [31]. Zwar ermöglichen die DaTraV-Daten aufgrund der hohen Fallzahl prinzipiell kleinräumige Analysen, jedoch müssen datenschutzrechtliche Aspekte gewahrt bleiben, um das Reidentifikationsrisiko der Versicherten zu minimieren. Mit Blick auf die in ReFern betrachteten in der Bevölkerung häufig vorkommenden NCD sollten Auswertungen auf kleinräumiger Ebene realisierbar sein, da hier mit ausreichend hohen Fallzahlen zu rechnen ist. Weitere Einschränkungen ergeben sich aus der schwankenden Dokumentationsqualität sowie der Nichterfassung der unerkannten Morbidität. Bei manchen Erkrankungen, wie z. B. Diabetes, vergehen häufig mehrere Jahre, bevor die Erkrankung ärztlich diagnostiziert wird – ein Zeitraum, in dem in der Regel schon Komplikationen auftreten. Insofern spielt neben dem bekannten (ärztlich diagnostizierten) Diabetes auch der unerkannte (ärztlich nicht diagnostizierte) Diabetes eine wichtige Rolle [32]. Informationen zu unerkannten Diabetes-Diagnosen liegen in den RKI-Untersuchungssurveys vor [33]. Insofern bilden Routinedaten eine sinnvolle Ergänzung zu den bundesweiten Gesundheitssurveys des RKI für die geplante NCD-Surveillance.

Basierend auf den inhaltlichen und praktischen Herausforderungen werden nutzerrelevante Anpassungen an der Analyseplattform innerhalb einer gesicherten Umgebung (Secure Processing Environment, SPE) für die DaTraV-Daten vorgenommen, um den Daten- und Ergebniszugang zu verbessern. Dabei ist wichtig, die Perspektive der nutzungsberechtigten Institutionen einzubinden und sich den Herausforderungen bei der Nutzung der DaTraV-Daten zu stellen. Das RKI bringt die Perspektive einer nutzungsberechtigten Institution in das Projekt ein und unterstützt somit aktiv das FDZ Gesundheit in seiner nutzerorientierten Weiterentwicklung.

## Fazit und Ausblick

Die Versorgungsdaten aller gesetzlich Krankenversicherten nach Datentransparenzverordnung, kurz DaTraV-Daten, bieten ein großes Potenzial für die sich im Aufbau befindende Surveillance nicht-übertragbarer Erkrankungen (NCD-Surveillance) am Robert Koch-Institut (RKI). Mit Expert:innen entwickelte standardisierte Referenzauswertungen können dabei unterstützen, in regelmäßigen Abständen regional und über die Zeit vergleichbare Schätzungen zu Prävalenz und Inzidenz wichtiger Public-Health-Erkrankungen bereitzustellen. Aufgrund der Tatsache, dass Routinedaten auch mit Limitationen verbunden sind, sollten diese im Kontext anderer Datenquellen, wie etwa Primärdaten, bewertet werden. Aufgrund der hohen Fallzahl bieten die DaTraV-Daten die Möglichkeit, zeitnah Informationen zu Morbidität auf kleinräumiger Ebene zu liefern, was insbesondere zur Planung und Umsetzung von Maßnahmen der Gesundheitsförderung und Prävention für gesundheitspolitische Akteur:innen sowohl auf Bundesebene als auch in Ländern sowie Kreisen und Kommunen relevant ist. Vor dem Hintergrund, dass gegenwärtig auf Basis von Gesundheitsstudien Daten zur Morbidität nur in großen zeitlichen Abständen verfügbar sind, können die DaTraV-Daten eine wichtige Informationslücke für eine kontinuierliche NCD-Surveillance schließen. Für die Umsetzung sind der Aufbau des Forschungsdatenzentrums Gesundheit (FDZ Gesundheit) am BfArM und die Realisierung eines Fernzugriffs auf die DaTraV-Daten in einer gesicherten Umgebung (SPE) von großer Bedeutung. Perspektivisch sollte eine automatisierte Durchführung der Referenzauswertungen verfolgt werden, um die Surveillance von NCD in Deutschland nachhaltig zu stärken.
